# What Would Next Generation Sequencing Bring to the Diagnosis and Treatment of Sarcomas? A Series of 20 Cases, a Single Institution’s Experience

**DOI:** 10.5146/tjpath.2021.01544

**Published:** 2021-09-15

**Authors:** İbrahim Kulaç, Pınar Bulutay, Çisel Aydın Meriçöz

**Affiliations:** Department of Pathology, Koç University, School of Medicine, Istanbul, Turkey

**Keywords:** Sarcoma, Soft tissue tumors, Next generation sequencing, Molecular pathology

## Abstract

***Objective:*** Soft tissue tumors comprise a small proportion of a pathologist’s routine practice. Although morphology and immunohistochemistry are quite helpful for diagnosing these tumors, many require molecular tests. Fluorescence in-situ hybridization has been the most commonly used method for the detection of specific genomic alteration, but next generation sequencing (NGS) could be more informative in many ways. Here we present our targeted NGS experience on soft tissue tumors with a series of 20 cases.

***Material and Method:*** The Laboratory Information System (LIS) was screened for soft tissue tumors that had been sequenced by NGS (between January 2018 - February 2021). 20 consecutive cases were included in the study. All cases were sequenced using a commercial targeted sequencing panel designed for soft tissue tumors.

***Results:*** We were able to run a reliable sequencing study for 16 (80%) of the cases but 4 (20%) of them failed in quality tests. We have found pathogenic alterations in 12 (60%) of the cases. The most common alterations were *EWSR1* fusions, *FLI1* being the most common partner. *NGS* results drastically changed the initial diagnosis, and thus the treatment modalities, in 3 cases (15%): the case with *ETV6-NTRK3* fusion, the case with *FUS-TFCP2* fusion, and the case of rhabdomyosarcoma (RMS) that was favored to be of the alveolar subtype and turned out to lack *FOXO1* fusions.

***Conclusion:*** A targeted NGS panel is robust and very informative. It not only allows pathologists to further specify and/or confirm their diagnosis but it could also play an important role in predicting the outcome.

## INTRODUCTION

Soft tissue sarcomas may occur at any age and at any localization. American Cancer Society estimates that more than 12 000 people will be diagnosed soft tissue sarcoma in 2021 (1). Although sarcomas seem to comprise only a small part of a pathologist’s daily practice, the diagnosis can often be challenging, time consuming and laborious. Many reports with inconclusive diagnosis that require molecular tests are commonly signed out inevitably.

Classification of these tumors has become more complicated and detailed after each WHO Classification of Tumors of Soft Tissue and Bones book edition (2). Hematoxylin & eosin (H&E) sections and a panel of immunohistochemistry with relevant antibodies with the guidance of a detailed radiological & clinical evaluation can be quite helpful for an accurate diagnosis. However, many entities have close resemblance at the H&E level and even their immunoexpressional profile is very similar. Molecular tests for a significant number of cases are usually needed and can be definitive. The key genetic event for soft tissue tumors is gene fusions and demonstrating those genetic alterations is not only diagnostic but may also have predictive value. There are a number of methods available today and most institutions prefer fluorescence in-situ hybridization (FISH) or reverse transcriptase PCR (RT-PCR).

FISH has been one of the most widely used techniques worldwide to detect gene re-arrangements (3,4). Traditionally, dual color and split signal FISH probes are used and allow investigations to detect the break of that gene, which is suggestive for a rearrangement (5). Although this approach is highly valuable, it usually lacks the information of the partner gene. Knowing the partner gene can be invaluable for the diagnostic dilemma such as seen with desmoplastic small round cell tumor (DSRCT) versus Ewing sarcoma since both might have fusions involving EWSR1 but with a different partner gene. There are other types of FISH probes that target each gene, and a fusion signal is suggestive for a rearrangement of the two. This approach is highly useful, although it provides no information on the exons involved. Another method is RT-PCR which aims to amplify the target cDNA converted from the RNA extracted from the tumor tissue (3,6). This approach is fast, cheap and can help to identify the specific exonal regions of the fusion. However, when the differential diagnosis includes numerous entities, all with different genomic alterations, it is substantially cumbersome to test all the specific alterations by FISH or RT-PCR for each one.

Massive parallel sequencing (next generation sequencing / NGS) has recently become a method of choice in many institutions for sequencing studies. It allows to sequence multiple genes at once with reasonable speed. cDNA synthesized from RNA or genomic DNA obtained from paraffin block can be used as the starting material. This technology has been used for years for cancer research and has more recently been introduced into clinical practice. Detection of cancer associated germline or somatic alterations has never been this practical. Although it still has a high price point and there are issues about the regulations and reimbursements, NGS is providing invaluable information about tumors.

Here we report a series of 20 soft tissue tumors that were evaluated by NGS for further genomic characterization using a commercial targeted NGS panel.

## MATERIAL and METHOD

### Case Selection

The Koç University Hospital, Department of Pathology’s Laboratory Information System (LIS) was screened for all soft tissue tumors that had been sequenced by NGS (between January 2018-February 2021). Among the 600 various NGS studies, it was found that 20 cases with a (proposed) diagnosis of soft tissue tumor were sequenced using the ArcherDx Sarcoma Panel. The ArcherDx sarcoma panel covers the most commonly altered genes in sarcomas (*ALK, BCOR, BRAF, CAMTA1, CIC, CSF1, EGFR, EPC1, ERG, ESR1, EWSR1, FGFR1, FGFR2, FGFR3, FOS, FOSB, FOXO1, FUS, GLI1, HMGA2, JAZF1, MDM2, MEAF6, MET, MGEA5, MKL2, NCOA1, NCOA2, NR4A3, NTRK1, NTRK2, NTRK3, NUTM1, PAX3, PDGFB, PHF1, PLAG1, PRKCA, PRKCB, PRKCD, RET, ROS1, SS18, STAT6, TAF15, TCF12, TFE3, TFG, USP6, YAP1, YWHAE*). Eleven of the cases were consults and 9 were from our department.

### Targeted Sequencing

Qiagen AllPrep DNA/RNA FFPE Kit was used for RNA extraction following the manufacturer’s protocol. RNA quantity was measured with the Qubit fluorometric quantification system (Life Technologies). Before library preparation, cDNA was synthesized from all RNA samples and a control PCR for quality assessment was performed. Although the library preparation kit requires a Ct value of <27, we had to include samples with Ct values of 27-30 because the cases did not have any other tissue samples. After the QC PCR run, cDNA library was prepared with the Archer FusionPlex Sarcoma kit (ArcherDX, Boulder, CO) following the manufacturer’s protocol. Libraries were run on the Illumina NextSeq 500 with compatible flow cells. All the analyses were done by using the ArcherDx Analysis software (version 6.2.7) and variants were confirmed with publicly available somatic variant databases.

## RESULTS

### Demographics and Basic Information About Cases/Samples

The median age of the patients was 20 (1-76) years. The female to male ratio was 9/11. We were unable to perform the study for four (20%) of the cases that had a Ct value of >30 at the QC PCR study. All 16 cases had acceptable read numbers and coverage. 11 (55%) of the cases were consults and 9 (45%) of them were from our department. 18 (90%) of the samples were paraffin blocks, 1 of the remaining two cases was a fresh frozen tissue stored in a -80°C freezer for a month after the surgery, and the other sample was a cell block from a mediastinal fine needle aspiration.

### Detected Alterations

We have found pathogenic alterations in 12 (60%) of the cases (Table 1). The most common alteration was *EWSR1-FLI1* fusion (three cases) and fusions involving *FOXO1 *(two cases). All the cases with *EWSR1-FLI1* fusion were referred to us for confirmation of the proposed Ewing sarcoma diagnosis.

**Table 1:** All the cases included to the study with their initial and final diagnosis along with genomic alterations detected by targeted NGS studies

**Initial Diagnosis**	**Alteration (Exons)**	**Final Diagnosis**
Ewing sarcoma	*EWSR1 – FLI1* (Ex 7 – Ex 6)	Ewing sarcoma
Ewing sarcoma	*EWSR1 – FLI1* (Ex 7 – Ex 6)	Ewing sarcoma
Ewing sarcoma	*EWSR1 – FLI1* (Ex 10 – Ex 5)	Ewing sarcoma
Clear cell sarcoma of the soft parts	*EWSR1 - CREM* (Ex 7 – Ex 7)	Clear cell sarcoma of the soft parts
Rhabdomyosarcoma, favor alveolar RMS	*PAX3 - FOXO1* (Ex 7 – Ex 2)	Alveolar RMS
Rhabdomyosarcoma, NOS	*PAX3 - FOXO1* (Ex 7 – Ex 2)	Alveolar RMS
Angiomatoid fibrous histiocytoma	*ETV6 - NTRK3* (Ex 5 – Ex 15)	Inflammatory myofibroblastic tumor / infantile fibrosarcoma
Sarcoma with epithelioid morphology	*TFCP2 - FUS* (Ex 2 – Ex 6)	Spindle and epithelioid rhabdomyosarcoma
Synovial sarcoma	*SS18 - SSX2* (Ex 9 – Ex 6)	Synovial sarcoma
Solitary fibrous tumor	*NAB2 *-* STAT6 *(Ex 6 – Ex 16)	Solitary fibrous tumor
PEComa	*SFPQ *- *TFE3* (Ex 9 – Ex 5)	PEComa
MUC4 Negative sclerosing epithelioid fibrosarcoma	*YAP1 - KMT2A* (Ex 7 – Ex 6)	MUC4 Negative sclerosing epithelioid fibrosarcoma
Rhabdomyosarcoma, favor embryonal RMS	No Alteration Detected	Embryonal RMS
Rhabdomyosarcoma, favor alveolar RMS	No Alteration Detected	Embryonal RMS
Undifferentiated sarcoma	No Alteration Detected	N/A
Small round cell tumor	No Alteration Detected	N/A
PEComa	N/A	N/A
High grade malignant mesenchymal tumor	N/A	N/A
Rhabdomyosarcoma, NOS	N/A	N/A
Embryonal sarcoma of the liver	N/A	N/A

**RMS:** Rhabdomyosarcoma, **NOS:** Not Otherwise Specified, **N/A:** Not applicable.

NGS results drastically changed the initial diagnosis, and thus the treatment modalities, in 3 cases (15%): the case with *ETV6-NTRK3* fusion, the case with *FUS-TFCP2* fusion, and the case of rhabdomyosarcoma (RMS) that was favored to be of the alveolar subtype and turned out to lack *FOXO1* fusions.

Two (10%) of the cases had no specific diagnosis other than high-grade malignant mesenchymal tumor and undifferentiated sarcoma, and we were unable to detect any specific alterations in these two tumors.

## DISCUSSION

Although soft tissue tumors comprise only a small part of a pathologist’s routine practice, cases can sometimes be diagnostically challenging. In adults, liposarcoma and leiomyosarcoma are among the most common sarcoma types and most of the time do not require any further studies other than H&E evaluation and immunohistochemistry. However, other relatively common tumors such as rhabdomyosarcoma and Ewing’s sarcoma/PNET often need the identification of the pathognomonic translocation because they have overlapping features with several entities and immunohistochemistry could be helpful only at a certain level. In daily practice we also encounter some rare soft tissue tumors that have less known but specific genetic alterations. These alterations can be investigated by techniques such as FISH, RT-PCR and NGS. They all have advantages and disadvantages. FISH and RT-PCR are tests that require testing for each gene with a separate reaction. They can be very helpful for some cases. However, especially when the morphology is vague and the immunohistochemistry does not direct towards a specific diagnosis, it would be hard to test the tumor tissue for every possible alteration with FISH and RT-PCR. In that case an NGS with a targeted panel would help substantially. There are two major approaches to hunt gene fusions by NGS that mainly differ by the starting material; the genomic DNA-based approach and the RNA-based approach. A RNA-based application enables the detection of fusions and even rare genomic abnormalities with high confidence if the isolated nucleic acid’s quality is high enough. There are several in-house and commercial targeted NGS panels that are designed for different sequencing platforms; one we have been using and reported in this study is RNA based and has a good coverage for many genes related to soft tissue tumors.

There are two major aspects of this study, and we would like to discuss them separately:

### Common Fusions That Require the Identification of Fusion Partners

Ewing sarcoma can overlap with many similar entities, especially with desmoplastic small round cell tumor (DSRCT), morphologically, clinically, and immunohistochemically; both are tumors of children and young adults, and although DSRCT is commonly located intraabdominally it can be encountered anywhere, as can Ewing sarcoma (7). For an experienced soft tissue pathologist, this differential may not be an issue for most of the cases using relevant morphological clues and some immunohistochemistry studies. Although WT1 is commonly used to differentiate these two and considered to be highly reliable (8) it may not be that helpful for all cases. A FISH study can be performed but a break-apart probe would only tell whether there is a rearrangement involving *EWSR1*. Using fusion-specific probes (for all possible genes) will increase the cost and time. A NGS panel that covers the most common genes rearranged in round cell sarcomas would be more efficient as regards cost and time. Moreover, undifferentiated round cell tumors of the bone and soft tissues have recently become more diverse. Genetically and clinically different round cell sarcomas were recently identified: round cell sarcoma with *EWSR1*-non ETS (*NFATC2* and *PATZ1* being the most common ones), CIC-rearranged sarcomas and sarcomas with *BCOR* genetic alteration. These tumors can have morphological features resembling Ewing sarcoma (9–11). Although these tumors share morphological similarities, they are distinct entities with different clinical and prognostic features. The differentiation is almost solely possible by a NGS panel that covers all the relevant genes. In our series, we had (non-RMS) 5 round cell tumors that were directed to molecular pathology for the detection of relevant alterations and all had *EWSR1-FLI1* fusions. Moreover, it was shown that different fusion transcripts may have various degrees of clinical impact for many neoplasms including Ewing sarcoma (12,13).

The detection of characteristic fusion is becoming a requirement for rhabdomyosarcomas as well. RMSs are histologically classified as alveolar, embryonal, pleomorphic, spindle cell/sclerosing with alveolar and embryonal RMSs being the most common types (2). In the past, the differentiation was made morphologically (with the help of immunohistochemistry) but for at least two decades the diagnosis is being supported by a FISH study using a *FOXO1* break-apart probe. The Children’s Oncology Group recently published a report that *FOXO1* fusion status was the most important prognosticator after metastatic status (14). Although it is very much possible to say that the tumor most likely to have this mutation by morphology, a FISH or a NGS study would be more helpful when needed.

### Rare Fusions in Rare Tumors Either for Diagnosis or for Treatment

**Figure 1:**
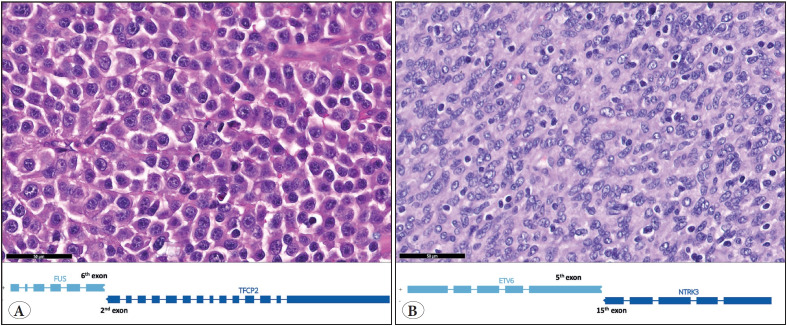
Hematoxylin & eosin sections of two selected cases. One (**A**) was initially called a malignant tumor with epithelioid morphology and considered an *EWSR1* non-ETV fusion sarcoma. An NGS study revealed a *TFCP2-FUS* fusion; the case was eventually diagnosed epithelioid and spindle rhabdomyosarcoma with *TFCP2-FUS* fusion. The other case (**B**) was initially called angiomatoid fibrous histiocytoma and NGS study revealed *ETV6-NTRK3* fusion. It was eventually diagnosed as

In our series we had three interesting and rare cases that deserve more attention. One of the cases was a molecular consult that was directed to our department for sequencing. The patient was a 33-year-old female, and her tumor was localized in the maxilla. The tumor was composed of epithelioid and spindle areas with extensive necrosis, and immunohistochemistry showed positivity with pancytokeratin, Desmin and MyoD1, which were all noted in an external pathology report. The differential included entities such as rhabdomyosarcoma and sarcomatoid carcinoma along with others and the case was reported as “sarcoma with epithelioid morphology” with a comment that said, “malignant tumor, favor *EWSR1-PATZ1* fusion sarcoma (EPS)” with a note: “sequencing was recommended”. Only one representative block was sent to our department for sequencing. A hematoxylin & eosin section of the block revealed an epithelioid/rhabdoid looking malignant tumor (Figure 1). A RNA based approach was used as described in detail in the material – methods section and the study revealed the *FUS-TFCP2* fusion. The case was diagnosed as “epithelioid and spindle cell rhabdomyosarcoma with *FUS-TFCP2* fusion” in accordance with the morphological, immunohistochemical and molecular findings (15,16).

Another patient was a 7-year-old male with a tumor at the pleura. The tumor looked spindly, cellular, and relatively monotonous (Figure 1). The initial report favored angiomatoid fibrous histiocytoma and the patient reached our department for sequencing. Our sequencing study revealed an *ETV6-NTRK3 *fusion. We have finalized the report by saying “infantile fibrosarcoma and inflammatory myofibroblastic tumor can be considered in the differential diagnosis with the morphological, immunohistochemical and molecular findings (17–19)”. Apart from the final diagnosis, this patient could be a candidate for targeted therapy because of this *NTRK3* fusion (20).

The last case was a 12-year-old boy with a mass in right thigh. The tumor was removed, and the specimen was evaluated at an outside pathology laboratory. The case was considered sclerosing epithelioid fibrosarcoma (SEF) although it was negative for MUC4, immunohistochemically. The patient was referred to our department for NGS studies. We have detected a fusion between *KMT2A* and *YAP1 *genes and this was supportive of the initial SEF diagnosis. SEFs are rare tumors harboring *EWSR1 *fusions commonly with *CREB3L1* gene. Recent studies suggested that there is a group of tumors with *KMT2A-YAP1* fusion that have almost identical morphological features with SEF but without the MUC4 positivity and *EWSR1* fusion. So far, researchers have called these tumors MUC4-negative SEF (21,22). The data is limited on these tumors, and it is not well documented whether these genomic alterations have different impact on prognosis. We think that, this clarification will most likely be possible when a sufficient number of cases are reported with their detailed clinical and morpho-molecular workup.

Detailed morphological evaluation and extensive immunohistochemical studies were performed on all three tumors discussed above. Today’s knowledge on soft tissue tumors makes us more aware of rare entities with specific genetic alterations, which entails us to perform molecular studies. Although molecular findings would not add any prognostic or predictive information for some tumors, it would have a huge impact in some. There are several publications on the utility and the benefits of a targeted NGS for soft tissue sarcomas (23–25).

NGS may seem like a highly advantageous technique especially in the context of gene fusions, but it is rather long and still costs a lot. Although we think that NGS will overtake many techniques in the future, we cannot deny the fact that the use of techniques such as RT-PCR and FISH is currently more practical for many patients. Finally, we would like to emphasize that NGS is only an ancillary technique like FISH or RT-PCR. They all need to be used in conjunction with microscopic evaluation and immunohistochemistry and need to be interpreted with caution by a pathologist. Although not within the scope of this paper, we would like to indicate that preanalytical variables are extremely important and all the samples should be fixed and stored with care. A last word should be said on the interpretation of NGS results, as they should be interpreted according to bioinformatic metrics and one should take coverage, number of reads and other quality parameters into consideration.

In conclusion, a RNA based NGS approach is highly valuable for the diagnosis of soft tissue tumors especially while dealing with rare cases with less known genomic alterations. This technique allows pathologists to further specify and/or confirm their diagnosis while providing predictive outcomes in some cases. Results of an NGS study should carefully be evaluated with clinical, histomorphological and immunohistochemical findings by the pathologist to finalize the case, in the best way possible.

## Conflict of INTEREST

The authors declare that they have no conflict of interest.

## References

[ref-1] Siegel Rebecca L., Miller Kimberly D., Fuchs Hannah E., Jemal Ahmedin (2021). Cancer Statistics, 2021. CA Cancer J Clin.

[ref-2] Board WHO Classification of Tumours Editorial (2020). Soft Tissue and Bone Tumours: WHO Classification of Tumours.

[ref-3] DuBois Steven G., Krailo Mark D., Buxton Allen, Lessnick Stephen L., Teot Lisa A., Rakheja Dinesh, Crompton Brian D., Janeway Katherine A., Gorlick Richard G., Glade-Bender Julia (2021). Patterns of Translocation Testing in Patients Enrolling in a Cooperative Group Trial for Newly Diagnosed Metastatic Ewing Sarcoma. Arch Pathol Lab Med.

[ref-4] Iwasaki Hiroshi, Nabeshima Kazuki, Nishio Jun, Jimi Shiro, Aoki Mikiko, Koga Kaori, Hamasaki Makoto, Hayashi Hiroyuki, Mogi Ai (2009). Pathology of soft-tissue tumors: daily diagnosis, molecular cytogenetics and experimental approach. Pathol Int.

[ref-5] Miura Yasuhiro, Keira Yoshiko, Ogino Jiro, Nakanishi Katsuya, Noguchi Hiroko, Inoue Tomomi, Hasegawa Tadashi (2012). Detection of specific genetic abnormalities by fluorescence in situ hybridization in soft tissue tumors. Pathol Int.

[ref-6] Castagnetti Fausto, Gugliotta Gabriele, Breccia Massimo, Iurlo Alessandra, Levato Luciano, Albano Francesco, Vigneri Paolo, Abruzzese Elisabetta, Rossi Giuseppe, Rupoli Serena, Cavazzini Francesco, Martino Bruno, Orlandi Ester, Pregno Patrizia, Annunziata Mario, Usala Emilio, Tiribelli Mario, Sica Simona, Bonifacio Massimiliano, Fava Carmen, Gherlinzoni Filippo, Bocchia Monica, Soverini Simona, Bochicchio Maria Teresa, Cavo Michele, Giovanni Martinelli, Saglio Giuseppe, Pane Fabrizio, Baccarani Michele, Rosti Gianantonio, GIMEMA CML Working Party (2017). The BCR-ABL1 transcript type influences response and outcome in Philadelphia chromosome-positive chronic myeloid leukemia patients treated frontline with imatinib. Am J Hematol.

[ref-7] Sbaraglia Marta, Righi Alberto, Gambarotti Marco, Dei Tos Angelo P. (2020). Ewing sarcoma and Ewing-like tumors. Virchows Arch.

[ref-8] Hill D. A., Pfeifer J. D., Marley E. F., Dehner L. P., Humphrey P. A., Zhu X., Swanson P. E. (2000). WT1 staining reliably differentiates desmoplastic small round cell tumor from Ewing sarcoma/primitive neuroectodermal tumor. An immunohistochemical and molecular diagnostic study. Am J Clin Pathol.

[ref-9] Antonescu Cristina R., Owosho Adepitan A., Zhang Lei, Chen Sonja, Deniz Kemal, Huryn Joseph M., Kao Yu-Chien, Huang Shih-Chiang, Singer Samuel, Tap William, Schaefer Inga-Marie, Fletcher Christopher D. (2017). Sarcomas With CIC-rearrangements Are a Distinct Pathologic Entity With Aggressive Outcome: A Clinicopathologic and Molecular Study of 115 Cases. Am J Surg Pathol.

[ref-10] Kao Yu-Chien, Owosho Adepitan A., Sung Yun-Shao, Zhang Lei, Fujisawa Yumi, Lee Jen-Chieh, Wexler Leonard, Argani Pedram, Swanson David, Dickson Brendan C., Fletcher Christopher D. M., Antonescu Cristina R. (2018). BCOR-CCNB3 Fusion Positive Sarcomas: A Clinicopathologic and Molecular Analysis of 36 Cases With Comparison to Morphologic Spectrum and Clinical Behavior of Other Round Cell Sarcomas. Am J Surg Pathol.

[ref-11] Michal Michael, Rubin Brian P., Agaimy Abbas, Kosemehmetoglu Kemal, Rudzinski Erin R., Linos Konstantinos, John Ivy, Gatalica Zoran, Davis Jessica L., Liu Yajuan J., McKenney Jesse K., Billings Steven D., Švajdler Marián, Koshyk Olena, Kinkor Zdeněk, Michalová Květoslava, Kalmykova Antonina V., Yusifli Zarifa, Ptáková Nikola, Hájková Veronika, Grossman Petr, Šteiner Petr, Michal Michal (2021). EWSR1-PATZ1-rearranged sarcoma: a report of nine cases of spindle and round cell neoplasms with predilection for thoracoabdominal soft tissues and frequent expression of neural and skeletal muscle markers. Mod Pathol.

[ref-12] Alava E., Kawai A., Healey J. H., Fligman I., Meyers P. A., Huvos A. G., Gerald W. L., Jhanwar S. C., Argani P., Antonescu C. R., Pardo-Mindan F. J., Ginsberg J., Womer R., Lawlor E. R., Wunder J., Andrulis I., Sorensen P. H., Barr F. G., Ladanyi M. (1998). EWS-FLI1 fusion transcript structure is an independent determinant of prognosis in Ewing's sarcoma. J Clin Oncol.

[ref-13] Bieg Matthias, Moskalev Evgeny A., Will Rainer, Hebele Simone, Schwarzbach Matthias, Schmeck Sanja, Hohenberger Peter, Jakob Jens, Kasper Bernd, Gaiser Timo, Ströbel Philip, Wardelmann Eva, Kontny Udo, Braunschweig Till, Sirbu Horia, Grützmann Robert, Meidenbauer Norbert, Ishaque Naveed, Eils Roland, Wiemann Stefan, Hartmann Arndt, Agaimy Abbas, Fritchie Karen, Giannini Caterina, Haller Florian (2021). Gene Expression in Solitary Fibrous Tumors (SFTs) Correlates with Anatomic Localization and NAB2-STAT6 Gene Fusion Variants. Am J Pathol.

[ref-14] Hibbitts Emily, Chi Yueh-Yun, Hawkins Douglas S., Barr Frederic G., Bradley Julie A., Dasgupta Roshni, Meyer William H., Rodeberg David A., Rudzinski Erin R., Spunt Sheri L., Skapek Stephen X., Wolden Suzanne L., Arndt Carola A. S. (2019). Refinement of risk stratification for childhood rhabdomyosarcoma using FOXO1 fusion status in addition to established clinical outcome predictors: A report from the Children's Oncology Group. Cancer Med.

[ref-15] Chrisinger John S. A., Wehrli Bret, Dickson Brendan C., Fasih Samir, Hirbe Angela C., Shultz David B., Zadeh Gelareh, Gupta Abha A., Demicco Elizabeth G. (2020). Epithelioid and spindle cell rhabdomyosarcoma with FUS-TFCP2 or EWSR1-TFCP2 fusion: report of two cases. Virchows Arch.

[ref-16] Le Loarer François, Cleven Arjen H. G., Bouvier Corinne, Castex Marie-Pierre, Romagosa Cleofe, Moreau Anne, Salas Sébastien, Bonhomme Benjamin, Gomez-Brouchet Anne, Laurent Camille, Le Guellec Sophie, Audard Virginie, Giraud Antoine, Ramos-Oliver Irma, Cleton-Jansen Anne-Marie, Savci-Heijink Dilara C., Kroon Herman M., Baud Jessica, Pissaloux Daniel, Pierron Gaëlle, Sherwood Anand, Coindre Jean Michel, Bovée Judith V. M. G., Larousserie Frédérique, Tirode Franck (2020). A subset of epithelioid and spindle cell rhabdomyosarcomas is associated with TFCP2 fusions and common ALK upregulation. Mod Pathol.

[ref-17] Suurmeijer Albert J., Dickson Brendan C., Swanson David, Zhang Lei, Sung Yun-Shao, Huang Hsuan-Ying, Fletcher Christopher D., Antonescu Cristina R. (2019). The histologic spectrum of soft tissue spindle cell tumors with NTRK3 gene rearrangements. Genes Chromosomes Cancer.

[ref-18] Gatalica Zoran, Xiu Joanne, Swensen Jeffrey, Vranic Semir (2019). Molecular characterization of cancers with NTRK gene fusions. Mod Pathol.

[ref-19] Davis Jessica L., Lockwood Christina M., Albert Catherine M., Tsuchiya Karen, Hawkins Douglas S., Rudzinski Erin R. (2018). Infantile NTRK-associated Mesenchymal Tumors. Pediatr Dev Pathol.

[ref-20] Huang Franklin W., Feng Felix Y. (2019). A Tumor-Agnostic NTRK (TRK) Inhibitor. Cell.

[ref-21] Kao Yu-Chien, Lee Jen-Chieh, Zhang Lei, Sung Yun-Shao, Swanson David, Hsieh Tsung-Han, Liu Yun-Ru, Agaram Narasimhan P., Huang Hsuan-Ying, Dickson Brendan C., Antonescu Cristina R. (2020). Recurrent YAP1 and KMT2A Gene Rearrangements in a Subset of MUC4-negative Sclerosing Epithelioid Fibrosarcoma. Am J Surg Pathol.

[ref-22] Warmke Laura M., Meis Jeanne M. (2021). Sclerosing Epithelioid Fibrosarcoma: A Distinct Sarcoma With Aggressive Features. Am J Surg Pathol.

[ref-23] Lam Suk Wai, Cleton-Jansen Anne-Marie, Cleven Arjen H. G., Ruano Dina, Wezel Tom, Szuhai Karoly, Bovée Judith V. M. G. (2018). Molecular Analysis of Gene Fusions in Bone and Soft Tissue Tumors by Anchored Multiplex PCR-Based Targeted Next-Generation Sequencing. J Mol Diagn.

[ref-24] Italiano Antoine (2018). Is There Value in Molecular Profiling of Soft-Tissue Sarcoma?. Curr Treat Options Oncol.

[ref-25] Dickson Brendan C., Swanson David (2019). Targeted RNA sequencing: A routine ancillary technique in the diagnosis of bone and soft tissue neoplasms. Genes Chromosomes Cancer.

